# Dietary Modulation of the Enteric Nervous System: From Molecular Mechanisms to Therapeutic Applications

**DOI:** 10.3390/nu17223519

**Published:** 2025-11-11

**Authors:** Xintong Wang, Wen Zhang, Huihui Wang, Yuzhen Zhao, Pengjie Wang, Ran Wang, Yanan Sun, Fazheng Ren, Yixuan Li

**Affiliations:** 1 Key Laboratory of Precision Nutrition and Food Quality, Department of Nutrition and Health, China Agricultural University, Beijing 100083, China; xtwang@cau.edu.cn (X.W.); zhangwen02919@126.com (W.Z.); whhlyh1227@163.com (H.W.); 18735271315@163.com (Y.Z.); 15153515695@163.com (Y.S.); 2 Food Laboratory of Zhongyuan, Luohe 462300, China; wpj1019@cau.edu.cn (P.W.); wangran@cau.edu.cn (R.W.)

**Keywords:** enteric nervous system, foodborne, myenteric plexus, submucosal plexus

## Abstract

The enteric nervous system (ENS), frequently referred to as the “second brain,” is integral to maintaining gastrointestinal and systemic homeostasis. The structural and functional homeostasis of the ENS is crucial for both local intestinal processes (digestion, immunity) and systemic physiological equilibrium via the gut–brain axis, directly influencing overall health and disease. In recent years, dietary substances have attracted increasing scholarly attention for their potential to modulate the ENS, attributed to their safety and accessibility. This review commences with a systematic exploration of the anatomical structure of the ENS, including the myenteric and submucosal plexuses, its cellular constituents such as enteric neurons and enteric glial cells, and its core physiological functions, encompassing the regulation of gastrointestinal motility, the secretion–absorption balance, and the maintenance of immune homeostasis. Subsequently, it delineates the classification, distribution, and properties of essential dietary components, encompassing polyphenols, short-chain fatty acids, amino acids and their derivatives, as well as prebiotics and probiotics. Additionally, it examines the mechanisms through which these substances modulate the physiological functions of the ENS, including the regulation of intestinal motility, support for neuronal survival and network integrity, and the maintenance of neuro-immune homeostasis. The review concludes by highlighting current limitations—including reliance on rodent models, unclear human ENS mechanisms, and imprecise interventions—and proposes future directions focused on precision medicine, clinical translation, and advanced tools like single-cell sequencing and targeted delivery systems.

## 1. Introduction

Among the body’s hollow organs, the gastrointestinal tract uniquely possesses a fully developed nervous system, termed the enteric nervous system (ENS), which is capable of functioning independently of neural inputs from the central nervous system (CNS), comprising the brain and spinal cord [1]. Structurally, the ENS is not a solitary neural network; rather, it consists of two interconnected plexuses: the myenteric plexus and the submucosal plexus. The myenteric plexus is primarily responsible for regulating intestinal motility, whereas the submucosal plexus predominantly governs mucosal secretion and absorption. A variety of enteric neurons and enteric glial cells (EGCs) within these plexuses collaborate to sustain ENS functionality [2]. Enteric neurons facilitate communication through a range of neurotransmitters, including cholinergic, nitrergic, and peptidergic mediators. EGCs contribute to ENS homeostasis by releasing neurotrophic factors, such as glial cell line-derived neurotrophic factor (GDNF), modulating neurotransmitter metabolism, and maintaining the integrity of the intestinal barrier [3]. Nevertheless, this intricate network is vulnerable to both endogenous and exogenous disturbances. Physiological aging may result in decreased neuronal density and imbalances in neurotransmitter release within the ENS [4]. Moreover, the integrity of the ENS is susceptible to compromise from its earliest developmental stages. Prenatal and early postnatal challenges—such as abnormalities in the migration of neural crest cells, mechanical constraints during the development of the abdominal cavity, disruptions in vascular supply, or birth trauma resulting in ischemia—can lead to congenital or acquired defects in the architecture and function of the ENS [5,6,7,8]. Under pathological conditions, such as chronic colitis or Parkinson’s disease (PD), factors like oxidative stress, excessive production of inflammatory cytokines, or metabolic disturbances in the gut microbiota can further exacerbate damage to the ENS. Consequently, these damages can lead to impaired intestinal motility, immune dysregulation, and may even promote pathological signaling from the periphery to the central nervous system via the gut–brain axis [9,10].

In recent years, dietary compounds have attracted considerable scholarly interest for their potential to modulate the ENS, owing to their favorable safety profiles and widespread availability [11]. Polyphenols, such as quercetin, have been shown to exert protective effects on the ENS by mitigating neuroinflammation [12]. Short-chain fatty acids (SCFAs), which are metabolites produced by the gut microbiota, not only serve as preferred energy sources for ENS neurons but also regulate neural signaling through receptors such as GPR43 and GPR41 [13]. Tryptophan and its metabolite serotonin (5-hydroxytryptamine, 5-HT) play crucial roles as neurotransmitters in ENS-mediated sensory-motor reflexes [14]. Probiotics and prebiotics contribute to the maintenance of intestinal homeostasis by modulating neurotransmitter release within the ENS and reshaping microbiota–ENS interactions [15,16]. While existing studies have made progress in elucidating the mechanisms of action of these dietary components, several significant limitations persist. Research models predominantly rely on rodents, and the cellular heterogeneity and regulatory mechanisms of the human ENS remain incompletely understood. Furthermore, targeted intervention strategies utilizing dietary substances in specific pathological contexts require further investigation. Additionally, the cooperative regulatory networks involving the ENS, CNS, and intestinal immune cells—such as feedback signaling mediated by the vagus nerve—are not yet fully comprehended.

In light of this, the present review systematically integrates the anatomical structure and cellular characteristics of the ENS and provides an in-depth analysis of the molecular mechanisms underlying its core physiological functions. Furthermore, it summarizes the regulatory pathways through which various dietary substances influence ENS physiology and their interventional effects in ENS-related disorders. This review aims to present a cohesive narrative by concentrating on the convergence of these dietary components on essential ENS processes—namely, neuroinflammation, neuronal survival, and neuro-immune communication. This focus will elucidate their influence on a range of conditions, from functional gastrointestinal disorders to neurodegenerative diseases, mediated through the gut–brain axis.

## 2. Enteric Nervous System

### 2.1. Anatomy and Structure of the Enteric Nervous System

A thorough understanding of gastrointestinal (GI) anatomy is crucial for studying the ENS distribution. The GI system includes the oral cavity, pharynx, esophagus, stomach, small intestine (duodenum, jejunum, and ileum), and large intestine (cecum, colon, and rectum) [17]. Key functions such as digestion, absorption, secretion, and motility occur mainly in the stomach, small intestine, and large intestine [17]. Histologically, the GI tract is composed of layers: lumen, epithelium, lamina propria, submucosa, muscularis externa, serosa, and mesentery [17]. The lumen hosts ingested nutrients and gut microbiota, which break down food into molecules absorbed by intestinal epithelial cells into the lamina propria [18]. The lamina propria contains connective tissue for structural support and numerous innate and adaptive immune cells. The structure is continuous with the submucosa, which serves as a conduit for blood vessels, neurons, and glial cells. The submucosa, in turn, is connected to the muscular layer and contains the submucosal plexus of the ENS. The muscularis externa is composed of an inner circular muscle layer and an outer longitudinal muscle layer, with the myenteric plexus situated between these two layers of smooth muscle. The outermost layers, the serosa and mesentery, envelop and lubricate the GI tract, thereby facilitating smooth contractile movements [19].

The mammalian nervous system is categorized into the CNS and the peripheral nervous system (PNS) [20]. The CNS encompasses the brain and spinal cord, whereas the PNS comprises all peripheral nerves [20]. Within the PNS, the autonomic nervous system (ANS) is responsible for regulating involuntary visceral functions without conscious oversight. The ANS is further divided into the sympathetic nervous system, the parasympathetic nervous system, and the ENS [21]. The human ENS contains approximately 500 million neurons, making it the largest aggregation of neurons outside the CNS. By autonomously regulating digestion, contributing to emotional processing, and maintaining immune homeostasis, the ENS functions as a critical nexus linking physiological and psychological health [22,23].

The myenteric plexus, also referred to as Auerbach’s plexus, forms a continuous, chain-like network that spans from the esophagus to the rectum [23]. Neuronal density within this plexus varies across different regions of the intestine, with the highest density found in the small intestine and colon, where approximately 1000 to 5000 neurons per square millimeter are present [24]. The primary role of the myenteric plexus is to regulate the contraction and relaxation of GI smooth muscles. The neuronal composition is predominantly comprised of motor neurons and interneurons, with a smaller proportion of sensory neurons [25]. Morphologically, the neurons within the myenteric plexus are mainly multipolar, characterized by extensive dendritic arbors that intricately interconnect. Their axons run parallel to the muscle layers, forming a dense network of nerve fibers that directly innervate smooth muscle cells, thereby modulating their activity [25]. Furthermore, the myenteric plexus is populated with numerous EGCs that envelop the neurons, forming a supportive framework similar to that of astrocytes in the CNS. These EGCs offer crucial trophic and metabolic support to the neuronal population [26].

The submucosal plexus, also referred to as Meissner’s plexus, is located between the GI mucosa and the circular muscle layer. Its distribution is relatively more limited compared to the myenteric plexus, being sparsely present in areas such as the esophagus and stomach, while being highly developed within the submucosa of the small and large intestines [27]. In contrast to the myenteric plexus, the submucosal plexus has a lower neuronal density, approximately 500–2000 neurons per square millimeter, but exhibits greater cellular diversity, primarily consisting of sensory neurons, motor neurons, and interneurons [24]. Among these, sensory neurons are the most prevalent. Their dendrites extend into the intercellular spaces of the intestinal epithelium or around crypts, enabling the direct detection of chemical signals (e.g., concentrations of fatty acids and amino acids), mechanical stimuli (e.g., luminal distension pressure), and osmotic changes within the intestinal lumen. Motor neurons primarily innervate glandular cells, epithelial cells, and vascular smooth muscle within the mucosa and submucosa, thereby regulating intestinal secretion, absorption, and local blood flow. Interneurons facilitate the transmission of sensory signals to motor neurons, thereby establishing local reflex circuits [25]. EGCs located within the submucosal plexus are also integral to this process. In addition to offering structural and metabolic support to neurons, EGCs are essential for maintaining the integrity of the intestinal mucosal barrier and are involved in immune modulation through the secretion of cytokines and neurotrophic factors [26].

**Figure 1:**
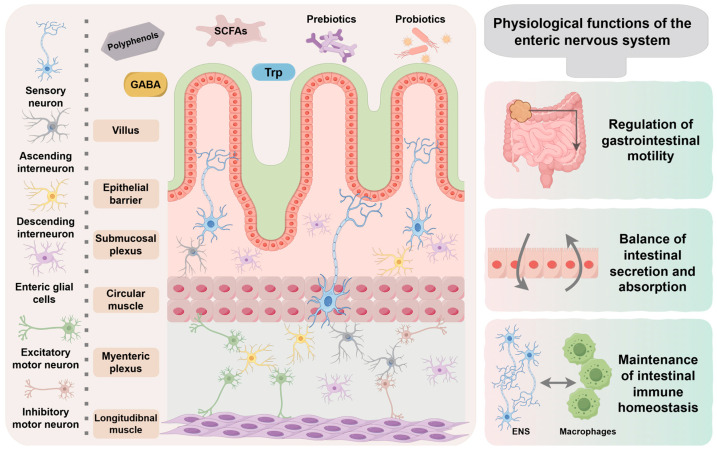
Schematic illustration of the enteric nervous system (ENS) components, structure, and physiological functions. This figure depicts ENS cell types, including sensory neurons, ascending interneurons, descending interneurons, enteric glial cells, excitatory motor neurons, and inhibitory motor neurons, alongside the structural organization of the ENS within the gastrointestinal tract (submucosal plexus, myenteric plexus, and their associations with epithelial barrier, villi, circular muscle, and longitudinal muscle). Moreover, molecular mediators and microbe-derived factors involved in modulating ENS activity (polyphenols, short-chain fatty acids (SCFAs), γ-aminobutyric acid (GABA), tryptophan, prebiotics, and probiotics) are illustrated, as well as key physiological functions regulated by the ENS: regulation of gastrointestinal motility, balance of intestinal secretion and absorption, and maintenance of intestinal immune homeostasis. This figure was drawn using FigDraw software (www.figdraw.com, accessed on 20 September 2025).

In conclusion, the architecture of the ENS is characterized by a complex, multi-layered network that spans from the mucosa to the serosa. Figure 1 provides a schematic representation of this intricate structure, detailing the exact positioning of the submucosal and myenteric plexuses within the intestinal wall. It also delineates their anatomical relationships with the smooth muscle layers and the epithelial barrier, thereby establishing a foundation for the ensuing analysis of their functional roles.

### 2.2. Cellular Composition of the ENS

#### 2.2.1. Enteric Neurons

The ENS constitutes a sophisticated cellular network, exhibiting a composition similar to that of the CNS. It predominantly consists of neurons and glial cells that are dispersed throughout the GI tract, where they oversee local functions and react to sensory stimuli originating both locally and from distant sources [25]. Neurons within the ENS are categorized according to various characteristics, such as morphology, neurotransmitter expression, cell surface receptors, electrophysiological properties, connectivity patterns, their specific location within the intestinal wall, and their functional roles.

Based on the morphological characteristics of ENS neurons, particularly the shape and length of their dendrites, Dogiel classified these neurons into types Dogiel I-III [28]. With the advent of advanced technologies, notably the application of single-cell RNA sequencing, a more intricate heterogeneity of neurons within the ENS has been uncovered. Through single-nucleus analysis of the human intestine, researchers have identified 436,202 nuclei and successfully isolated 1445 neurons [29]. The functionality of the ENS is dependent on the coordinated action of diverse neurotransmitters, which can be categorized according to their chemical properties and functions. Among cholinergic neurotransmitters, acetylcholine (ACh) is the most prevalent and plays a pivotal role in the ENS [22]. It is predominantly synthesized and released by excitatory motor neurons and certain interneurons. ACh binds to nicotinic acetylcholine receptors (nAChRs) on the postsynaptic membrane, initiating action potentials and subsequently inducing contraction of intestinal smooth muscle [30].

Monoaminergic neurotransmitters: Serotonin is a crucial monoamine neurotransmitter with dual roles within the ENS. It is released by enterochromaffin cells (ECs) and engages 5-HT3 receptors, a class of ionotropic receptors, to induce pronounced contractions of intestinal muscles [31]. Conversely, the activation of 5-HT4 receptors facilitates intestinal peristalsis and stimulates glandular secretion [32]. Additionally, 5-HT is integral to the modulation of intestinal inflammatory responses, with its dysregulation being strongly linked to inflammatory bowel disease (IBD) [33]. Amino acid neurotransmitters: Gamma-aminobutyric acid (GABA) functions as a major inhibitory neurotransmitter in the ENS, playing a critical role in maintaining intestinal immune homeostasis and regulating gut functions [34]. Peptidergic neurotransmitters play a crucial role in the ENS by mediating a variety of physiological functions. Vasoactive intestinal peptide (VIP), a key inhibitory neurotransmitter, is extensively distributed across the intestinal wall, with a particular concentration in the submucosal and mucosal layers. VIP exerts its effects by binding to specific receptors, resulting in the inhibition of smooth muscle contraction and a consequent decrease in intestinal motility [35]. Furthermore, VIP exhibits anti-inflammatory and immunomodulatory properties, which may have implications for the modulation of intestinal barrier function [36]. Substance P (SP), another significant peptidergic neurotransmitter, is predominantly found in the myenteric plexus and facilitates intestinal muscle contraction through its interaction with neurokinin-1 receptors [37]. Neuropeptide Y (NPY) also plays a role in modulating intestinal motility and secretion by inhibiting smooth muscle contraction and reducing glandular secretion [38]. In addition to peptidergic neurotransmitters, nitrogenic neurotransmitters such as nitric oxide (NO) are integral to ENS function. NO is a gaseous neurotransmitter synthesized primarily by nitric oxide synthase (NOS). It promotes smooth muscle relaxation and vasodilation through the activation of guanylate cyclase, which leads to an increase in intracellular cyclic guanosine monophosphate levels [39].

#### 2.2.2. EGCs

EGCs are critical components of the ENS and, in conjunction with enteric neurons, constitute a sophisticated neural network responsible for regulating a variety of gastrointestinal functions [40]. Initially identified by Dogiel in the late 19th century, EGCs were not formally characterized as specialized glial cells with distinct functional roles until the 1970s by Gabella [41]. In contrast to glial cells found in the central and peripheral nervous systems, EGCs possess unique morphological and functional attributes [42]. Within the ENS, EGCs are significantly more numerous than neurons, with ratios ranging from 3:1 to 10:1. They are extensively distributed throughout both the submucosal and myenteric plexuses, extending processes that form complex interaction networks with neurons, epithelial cells, immune cells, and the vascular system [43]. The morphology and function of EGCs are modulated by the local microenvironment, exhibiting considerable heterogeneity not only among different EGC subtypes but also in their distribution and functional specialization across various regions of the GI tract [26].

The heterogeneity of EGCs is predominantly manifested at three distinct levels: spatial, regional, and functional. Spatial heterogeneity pertains to the variations in morphology and function of EGCs within a specific intestinal region. For example, glial cells located in the myenteric plexus may display functional differences compared to those in the submucosal plexus [40]. Regional heterogeneity is evidenced by the differences in gene expression and functionality of EGCs across various segments of the GI tract, such as the small intestine and colon [44]. Recent single-cell transcriptomic analyses have identified substantial differences in gene expression profiles between EGCs in the human colon and small intestine, which likely reflect adaptations to region-specific functional requirements [45]. Additionally, the functional heterogeneity of EGCs is intricately linked to their local microenvironment. For instance, submucosal glial cells may play pivotal roles in regulating intestinal barrier function and immune responses, whereas intramuscular glial cells are primarily involved in modulating the motor function of intestinal smooth muscle [46]. This functional diversity enables EGCs to adapt effectively to their specific physiological contexts.

### 2.3. Core Physiological Functions of the ENS

The ENS serves as an autonomous regulatory center for GI functions. By coordinating the activities of the myenteric and submucosal plexuses, it facilitates precise regulation of digestion, secretion, absorption, and motility. The effective execution of these functions is contingent upon the homeostatic balance of the ENS, which is capable of operating independently of the CNS. Nonetheless, the ENS is concurrently subject to modulation by the CNS through the gut–brain axis, thereby establishing a dual regulatory mechanism characterized by local autonomy and central influence.

#### 2.3.1. Regulation of GI Motility

The ENS is integral to GI regulation, orchestrating the transit and mixing of intestinal contents to accommodate varying physiological conditions. During fasting, the GI tract remains relatively inactive, prompting the ENS to initiate the migrating motor complex (MMC) to maintain intestinal cleanliness and prepare for subsequent food intake [47]. The MMC is characterized by a cyclic, propulsive contractile pattern that originates in the gastric body and progresses along the small intestine toward the colorectum, with a periodicity of approximately 90–120 min. Its generation and regulation are predominantly mediated by the neuronal network within the myenteric plexus [47]. Following gastric emptying, cholinergic neurons within the myenteric plexus become activated, releasing ACh to induce intestinal contraction. Concurrently, nitrergic motor neurons release NO, which inhibits smooth muscle contraction in subsequent intestinal segments, thereby creating a lumen-opening effect that facilitates the propagation of the contractile wave. This alternating excitatory and inhibitory mechanism ensures the directional migration of the MMC [48].

The myenteric plexus plays a crucial role in the direct regulation of GI motility, whereas the submucosal plexus is essential for the perception of peristaltic stimuli. Upon the introduction of food into the GI tract, luminal distension and chemical signals activate sensory neurons within the submucosal plexus. This activation initiates ENS-mediated reflexes that modify intestinal motor patterns, resulting in a combination of segmental and peristaltic contractions [49]. Segmental contractions, which are primarily observed in the small intestine, are governed by local reflex circuits within the myenteric plexus. Conversely, peristaltic contractions, characterized by their propulsive nature, can occur throughout the entire gastrointestinal tract [50]. When chyme interacts with the intestinal wall, sensory neurons in the submucosal plexus transmit signals to interneurons located within the myenteric plexus. These interneurons, in turn, activate inhibitory motor neurons in the segment anterior to the chyme, leading to smooth muscle relaxation, and excitatory motor neurons in the segment posterior to it, resulting in smooth muscle contraction. This orchestrated pattern of “relaxation ahead and contraction behind” effectively facilitates the distal propulsion of chyme [51]. In response to fasting and feeding regimens, guinea pigs exhibit adaptive modifications in their myenteric neurons, which in turn produce specific gastrointestinal motility responses aligned with their nutritional state. Additionally, fasting-mimicking diets have been observed to retard tumor progression in murine models [52,53].

#### 2.3.2. Balance of Intestinal Secretion and Absorption

The regulation of the equilibrium between intestinal secretion and absorption represents a critical function of the ENS in sustaining internal homeostasis and enhancing nutrient absorption. This regulatory process is predominantly mediated by the submucosal plexus, which orchestrates the activities of intestinal epithelial cells, enteroendocrine cells, and vascular smooth muscle tissue [54]. In the context of secretion regulation, the ENS governs the release of digestive enzymes, mucus, and electrolytes, thereby establishing an optimal environment for food digestion [54]. Upon contact with intestinal epithelial cells, chemical constituents of food (e.g., fatty acids, amino acids) or microbial metabolites (e.g., SCFAs) trigger downstream signaling pathways, leading to the release of molecules such as 5-HT and ATP. These signaling molecules activate sensory neurons within the submucosal plexus, which subsequently—via interneurons—stimulate cholinergic motor neurons. The ACh released by these neurons acts on enteroendocrine cells, thereby promoting the secretion of digestive enzymes, mucus, and electrolytes [55].

In the regulation of absorption, ENS neurons influence the expression and activity of intestinal epithelial cells through neurotransmitter release [56]. Research indicates that ACh activates nicotinic receptors on intestinal epithelial cells, initiating calcium ion currents. These calcium signals propagate across the epithelial layer via gap junctions, facilitating cellular maturation while diminishing proliferation and inflammatory responses. Disruption of this process may result in chronic damage, characterized by ion imbalance, cell death, and an increase in inflammatory cytokines, akin to the pathological features observed in IBD [57].

#### 2.3.3. Maintenance of Intestinal Immune Homeostasis

The regulation of intestinal immune equilibrium constitutes a vital function of the ENS in sustaining gut homeostasis. Through bidirectional communication with immune cells, the ENS not only provides defense against pathogenic invasion but also mitigates excessive immune responses that could precipitate inflammation. This interaction involves intricate communication among ENS neurons, EGCs, and intestinal immune cells, including macrophages, dendritic cells, T cells, and mast cells [58]. Research has shown that helminth infection elevates intestinal levels of interferon-gamma (IFN-γ), which activates EGCs and stimulates the secretion of CXCL10. This chemokine subsequently recruits CD8^+^ T cells that produce additional IFN-γ, thereby facilitating IFN-γ-mediated tissue repair and damage control [59]. In a mouse model of colitis induced by dextran sulfate sodium (DSS), the ablation of choline acetyltransferase-positive (ChAT^+^) neurons aggravated colitis and resulted in motor dysfunction. In contrast, optogenetic activation of cholinergic neurons alleviated colitis, maintained smooth muscle contractility, prevented neuronal loss, and diminished the production of pro-inflammatory cytokines [60].

Immune cells are capable of negatively regulating ENS function through the secretion of cytokines and inflammatory mediators, thereby establishing a bidirectional immune-to-neuro signaling loop. In response to pathogen invasion or inflammatory stimuli, macrophages and dendritic cells secrete pro-inflammatory cytokines, including IL-6, IL-17, and TNF-α, which influence the activity of enteric neurons and EGCs [61]. With advancing age, macrophage polarization transitions from the M2 phenotype (anti-inflammatory) to the M1 phenotype (pro-inflammatory). This transition is associated with elevated cytokine levels and increased infiltration of immune cells within the ENS, which correlates with heightened apoptosis, neuronal and EGC loss, and delayed intestinal transit [62].

#### 2.3.4. Modulating Gut Microbiota Homeostasis

The interaction between the ENS and the gut microbiome constitutes a dynamic, bidirectional relationship. The microbiome significantly influences the development and function of the ENS, while the ENS, in turn, plays an active role in shaping the microbial ecosystem. Specifically, the ENS indirectly regulates microbial composition by modulating critical aspects of the intestinal environment. For instance, the ENS can affect the structure of microbial communities and regulate intestinal inflammatory responses by altering the pH of the microbial habitat [63]. In zebrafish with congenital agaglionosis resulting from mutations in the sox10 gene, which lead to the absence of an ENS, microbiota-dependent inflammation occurs. Restoration of ENS function in these genetically deficient zebrafish can ameliorate the disordered gut microbiota and pathological inflammation [64].

In contrast, the establishment of this bidirectional circuit occurs early in life. Recent research indicates that the diversification of the gut microbiota, which accompanies the postnatal maturation of the ENS, may play a critical role in the development and functionality of the ENS [10]. Studies have demonstrated that vaginal dysbiosis associated with Ureaplasma parvum infection leads to a reduction in enteric neurons and glial cells within the ovine fetal ENS [65]. The mode of delivery, whether vaginal or cesarean section, influences the initial microbial colonization of the infant; the gut microbiota of infants delivered vaginally more closely mirrors that of their mothers [66]. Additionally, breast milk, which influences the composition of the infant’s gut microbiota, contains neurotrophic factors and cytokines that support the survival and growth of enteric neurons in vitro [67].

Consequently, the microbiome–ENS interaction established during early life not only establishes the foundational developmental framework for intestinal physiology but also, when disrupted, predisposes individuals to a range of gastrointestinal and neurological disorders in later stages of life. This highlights the critical lifelong importance of maintaining homeostasis within this bidirectional axis.

## 3. Key Dietary Components for ENS Modulation and Their Properties

Dietary substances refer to naturally occurring compounds ingested through the daily diet that influence physiological functions and pathological processes in the body. These substances are derived from plant-based foods, animal products, and fermented foods. In the context of ENS regulation, dietary substances with clearly defined roles are not merely general nutrients but rather specific compounds that interact with the ENS through distinct pathways to modulate its structure and function.

### 3.1. Polyphenols

Polyphenols constitute a class of secondary metabolites synthesized by plants throughout evolutionary processes and are abundantly found in natural plant-based foods, including fruits, vegetables, nuts, legumes, tea, and herbal medicines. The defining structural characteristic of polyphenols is the presence of multiple phenolic hydroxyl groups (-OH) within their molecular structure. These compounds can be categorized into various subclasses based on their chemical structures, such as flavonoids, phenolic acids, stilbenes, and lignans. Among these, flavonoids represent not only the most diverse subclass but also the most extensively researched concerning their interactions with the ENS, comprising over 60% of total polyphenols [68]. The direct regulatory effects of polyphenols on the ENS are intricately linked to their distinctive physicochemical properties, intestinal metabolic behavior, and receptor-binding capabilities, rendering them a focal point in studies investigating the mechanisms by which dietary components influence the ENS.

The classification of polyphenols is predominantly determined by variations in their fundamental structural frameworks, with each subclass demonstrating unique specificity in its distribution across various food sources. These distribution patterns significantly affect the likelihood and quantity of specific polyphenol consumption among populations with diverse dietary practices. Flavonoids, identified by a C6–C3–C6 structure, can be further categorized into flavonols (e.g., quercetin, kaempferol), flavones (e.g., apigenin, luteolin), flavanones (e.g., naringenin, hesperetin), and chalcones, based on the oxidation state of the pyran ring and the nature of substituents [69]. Phenolic acids, derived from benzoic or cinnamic acid structures, primarily encompass hydroxybenzoic acids (e.g., gallic acid, *p*-hydroxybenzoic acid) and hydroxycinnamic acids (e.g., chlorogenic acid, caffeic acid) [70]. Stilbenes, characterized by a 1,2-diphenylethylene skeleton, are exemplified by resveratrol, which is predominantly found in grape skins, peanuts, and medicinal herbs such as Polygonum cuspidatum [71]. In studies related to ENS regulation, flavonoids have emerged as a major focus due to their strong affinity for ENS cellular receptors and their ability to modulate signaling pathways. Meanwhile, chlorogenic acid and resveratrol have also garnered significant attention for their notable anti-inflammatory, antioxidant, and gut–brain axis regulatory activities.

### 3.2. SCFAs

SCFAs are saturated fatty acids characterized by carbon chain lengths ranging from one to six carbons, predominantly comprising acetate, propionate, and butyrate, which collectively constitute over 95% of the total intestinal SCFAs. Unlike other dietary constituents, SCFAs are neither directly obtained from food intake nor synthesized endogenously by the human body. Their production is entirely contingent upon the fermentation of dietary fiber—particularly soluble fiber—by the gut microbiota [72]. The distribution of SCFAs within the intestine demonstrates a distinct regional gradient, closely linked to the sites of fermentation. The large intestine, especially the cecum and proximal colon, serves as the principal site for SCFA production due to its high microbial density and the abundance of fiber substrates, resulting in the highest concentrations of SCFAs. As intestinal contents progress toward the distal colon, the availability of substrates diminishes, leading to a gradual decline in SCFA levels. Conversely, SCFA concentrations in the stomach and small intestine are exceedingly low, primarily originating from the limited fermentation of undigested carbohydrates by the small intestinal microbiota [73]. The distribution pattern indicates that the effects of SCFAs on the ENS are primarily localized within the large intestine. Furthermore, SCFAs have the capacity to enter systemic circulation through the portal venous system, thereby exerting influence on distal intestinal regions and potentially affecting the CNS [73].

The absorption of SCFAs across the colonic mucosa is facilitated by two principal mechanisms. Over 90% of SCFAs are transported in their ionized form via active transporters, specifically monocarboxylate transporter 1 (MCT1) and sodium-coupled monocarboxylate transporter 1 (SMCT1), which are situated on the basolateral membrane of colonic epithelial cells. The remaining approximately 10% are absorbed through passive diffusion in their protonated form [74]. Once within the intestinal wall, butyrate is preferentially assimilated by colonic epithelial cells and ENS neurons. Epithelial cells utilize butyrate as an energy source through β-oxidation to produce ATP, whereas neurons use it to sustain metabolic functions and support neurotransmitter synthesis. In contrast, acetate and propionate predominantly enter the portal venous circulation to exert systemic effects. Thus, butyrate serves as a crucial SCFA in regulating ENS function, with its intestinal concentration directly affecting neuronal vitality and functional status within the ENS [74].

### 3.3. Amino Acids and Their Derivatives

#### 3.3.1. Tryptophan

Tryptophan is an essential aromatic amino acid distinguished by its β-carbon linked to the 3-position carbon of an indole ring. Among the 20 standard amino acids, tryptophan possesses the largest molecular mass and exhibits the lowest abundance in proteins and cells. Despite this scarcity, it functions as a crucial biosynthetic precursor for a diverse array of microbial and host metabolites, thereby imparting significant physiological importance. The capacity of certain microorganisms to synthesize tryptophan has been extensively harnessed in industrial applications. As cells are incapable of synthesizing tryptophan de novo, humans must acquire it from external sources, predominantly through dietary intake. Common natural dietary sources include oats, bananas, dried plums, milk, tuna, cheese, bread, poultry, peanuts, and chocolate [75].

Tryptophan is metabolized in vivo predominantly via three principal pathways: the kynurenine pathway, the serotonin pathway, and the gut microbiota-mediated metabolic pathway. The equilibrium among these pathways is crucial for the maintenance of systemic homeostasis [76]. The kynurenine (KYN) pathway constitutes the primary route for tryptophan catabolism, accounting for approximately 95% of total tryptophan degradation. The enzymes indoleamine 2,3-dioxygenase and tryptophan 2,3-dioxygenase serve as the rate-limiting factors in this pathway. This metabolic route yields various biologically active metabolites, including KYN, kynurenic acid (KYNA), and quinolinic acid (QA). Notably, KYNA possesses neuroprotective properties, whereas excessive levels of QA may lead to neuro-excitotoxicity [77]. The serotonin pathway is responsible for approximately 1–2% of tryptophan metabolism, with 5-HT as its primary end product. Serotonin is a crucial neurotransmitter involved in the regulation of mood, sleep, and appetite. Furthermore, 5-HT can be converted into melatonin, which plays a role in the regulation of circadian rhythms [78]. The microbial metabolic pathway is predominantly influenced by gut microbiota, leading to the production of indole derivatives, including indole-3-lactic acid (ILA) and indole-3-acetic acid (IAA). These metabolites have the capacity to modulate a range of host physiological processes through the activation of the aryl hydrocarbon receptor (AhR) [79].

#### 3.3.2. GABA

GABA is a naturally occurring non-protein amino acid that functions as the primary inhibitory neurotransmitter in the CNS and as a critical signaling molecule within the ENS [80]. In contrast to polyphenols, the regulatory impact of GABA on the ENS is characterized by neurotransmitter homology, as its chemical structure is identical to that of GABA endogenously synthesized and secreted by ENS neurons. Consequently, GABA can directly bind to GABA receptors in the ENS without necessitating structural modification or metabolic conversion, thereby exerting its physiological regulatory effects. This attribute positions GABA as one of the few dietary bioactive molecules capable of directly engaging in neural signaling within the ENS [81].

GABA is prevalent in a variety of plant-based foods and fermented products, with its concentration being significantly affected by the type of food, its maturity, and the methods of processing employed. Under conditions of stress, plants activate the enzyme glutamate decarboxylase (GAD), which catalyzes the conversion of glutamate to GABA, thereby enhancing stress tolerance. As a result, plant-based foods that undergo stress treatments, such as germinated grains and stress-exposed vegetables, frequently exhibit increased levels of GABA. Additionally, during the fermentation process, microorganisms, such as lactic acid bacteria, can express GAD, which converts glutamate in the substrate into GABA, thereby promoting its accumulation. This biochemical process renders fermented foods, including kimchi, yogurt, and cheese, significant dietary sources of GABA [80].

### 3.4. Prebiotics and Probiotics

#### 3.4.1. Prebiotics

Soluble dietary fiber encompasses a class of fibers that dissolve in water, forming gel-like substances. A key characteristic of soluble dietary fiber is its fermentability by gut microbiota, which positions it as a crucial element of prebiotics. By providing a carbon source for beneficial intestinal bacteria, soluble dietary fiber influences the composition and metabolic activity of the microbiota, thereby affecting ENS function via the microbiota–gut–brain axis [82]. These fibers are prevalent in plant-based foods and can be categorized based on their chemical structures into non-starch polysaccharides (such as inulin, pectin, and β-glucan), resistant oligosaccharides (such as fructooligosaccharides and galactooligosaccharides), and gums (such as guar gum and arabic gum). The diverse molecular structures of various soluble dietary fibers result in distinct selective regulatory effects on gut microbiota and fermentation properties [83]. The primary mechanism by which prebiotics exert their influence through the microbiota–gut–brain axis is rooted in the dual outcomes of their microbial fermentation. Firstly, prebiotics selectively enhance the growth of beneficial bacterial species, such as *Bifidobacterium* and *Lactobacillus*, thereby optimizing the microbial community structure. Secondly, the bioactive metabolites produced during fermentation, notably SCFAs, indirectly modulate the development and functionality of the ENS [82].

#### 3.4.2. Probiotics

Probiotics are live microorganisms that, when administered in sufficient quantities, provide health benefits to the host. Their regulatory effects on the ENS are entirely contingent upon the “microbiota–gut–brain axis” mechanism. This mechanism indirectly influences the structure and function of the ENS through intricate interactions among probiotics, gut microbiota, intestinal epithelial cells, and immune cells [15]. The mechanisms by which probiotics regulate the ENS via the microbiota–gut–brain axis can be delineated into three levels: microbial interactions, host cell interactions, and metabolic products. At the microbial interaction level, probiotics primarily enhance gut microbiota composition through strategies such as “competitive exclusion” and “microbial remodeling.” At the host cell interaction level, probiotics directly engage with intestinal epithelial cells, EGCs, and immune cells, secreting signaling molecules to modulate their functions, thereby affecting the ENS. At the metabolic level, bioactive metabolites produced by probiotics—including SCFAs, bacteriocins, vitamins, and indole derivatives—function as crucial signaling molecules that establish a connection between probiotics and the ENS [15,83]. This complex, multi-pathway regulatory mechanism highlights the substantial potential of probiotics in modulating ENS-related disorders, thereby positioning them as a promising strategy for dietary intervention.

### 3.5. Synergistic and Integrated Modulation of the ENS by Dietary Components

**Figure 2:**
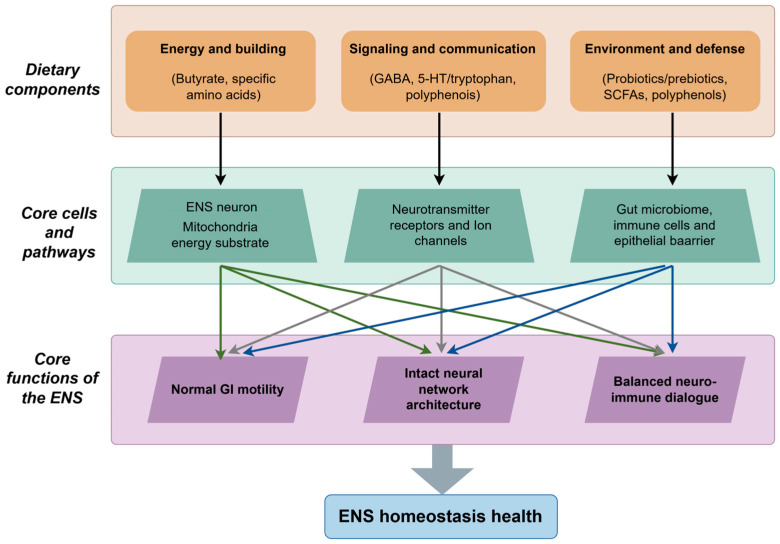
Schematic representation of the core components and pathways through which dietary factors regulate enteric nervous system (ENS) homeostasis. The figure categorizes essential dietary elements into three functional groups: (1) energy and building blocks (e.g., butyrate, specific amino acids), which serve as energy substrates for ENS neurons; (2) signaling and communication agents (e.g., GABA, 5-HT/tryptophan, polyphenols), which interact with neurotransmitter receptors and ion channels; and (3) environment and defense factors (e.g., probiotics/prebiotics, short-chain fatty acids (SCFAs), polyphenols), which engage with the gut microbiome, immune cells, and the epithelial barrier. Collectively, these components target fundamental cells and pathways to sustain the essential functions of the ENS, including the maintenance of normal gastrointestinal motility, the integrity of neural network architecture, and a balanced neuro-immune dialogue, thereby promoting ENS homeostasis and overall health. This figure was generated using FigDraw.

Despite their diverse origins and structures, dietary components do not independently regulate the ENS; rather, they converge significantly on a limited set of core physiological pathways. Through network-based regulation, these dietary components can exert synergistic or complementary effects, collectively sustaining ENS homeostasis. In the context of energy and nutrition, this axis is primarily concerned with providing essential metabolic support and stress protection for ENS cells. Key components include butyrate and other SCFAs, which serve as primary energy substrates, and polyphenols, which act as major regulators of oxidative homeostasis. In terms of neuromodulation, this aspect involves the direct regulation of intestinal neural signaling and subsequent gut motility. Such neuromodulation is profoundly influenced by microbially and diet-derived GABA as well as 5-HT/tryptophan metabolism, which precisely modulate the excitatory-inhibitory balance within ENS circuitry. With respect to the microenvironment and immune function, this component is integral to sustaining a healthy intestinal lumen and mucosal environment, which are essential for the proper functioning of the ENS. The regulation of this microenvironment and immune axis is predominantly facilitated by probiotics, prebiotics, and their metabolites. These elements collectively influence the composition of the gut microbiota and modulate neuro-immune interactions (Figure 2).

## 4. Modulatory Effects of Dietary Components on ENS Functions

**Figure 3:**
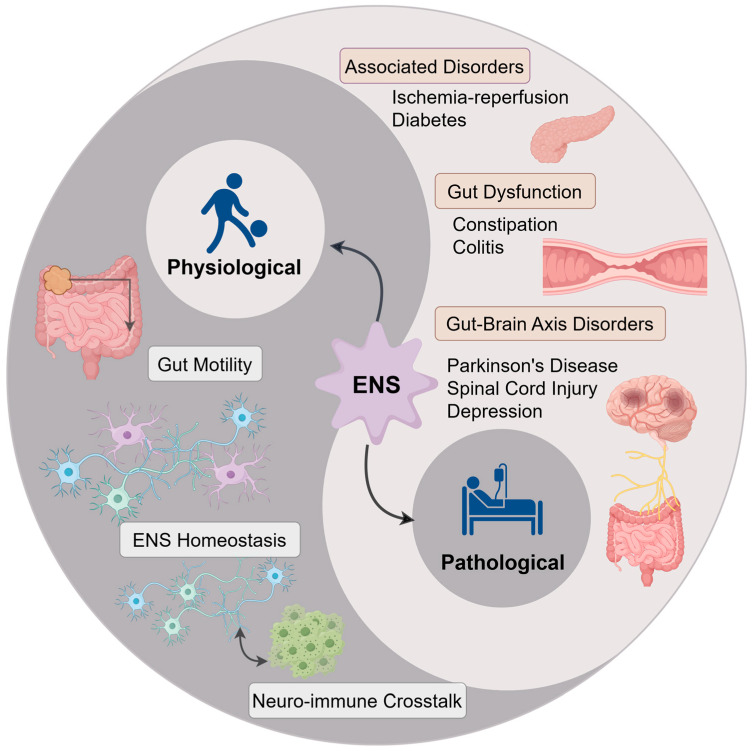
Schematic representation of the enteric nervous system (ENS) in physiological processes and pathological conditions. This figure illustrates the dual roles of ENS: the physiological section depicts key processes including gut motility, ENS homeostasis, and neuro-immune crosstalk; the pathological section summarizes ENS-related disorders, encompassing associated systemic conditions (ischemia–reperfusion, diabetes), gut dysfunction (constipation, colitis), and gut–brain axis disorders (Parkinson’s disease, spinal cord injury, depression). This figure was drawn using FigDraw software.

The ENS plays a crucial role in maintaining gastrointestinal homeostasis through the coordination of diverse cellular activities. As depicted in the physiological section of Figure 3, the fundamental functions of the ENS can be categorized into three interconnected pillars: the regulation of gastrointestinal motility, the maintenance of intrinsic homeostasis (encompassing neuronal and network integrity), and the facilitation of neuro-immune communication. The subsequent sections will explore the impact of dietary components on these three essential aspects.

### 4.1. Regulation of Intestinal Motility

Intestinal motility represents a fundamental physiological function of the ENS, which is dependent on the coordinated interactions among the motor neurons of the myenteric plexus, intestinal smooth muscle, and interstitial cells of Cajal. Dietary components have the capacity to precisely modulate intestinal contractility, peristaltic frequency, and slow wave potentials through three principal mechanisms: activation of receptor-mediated signaling pathways, modulation of neurotransmitter release, and provision of energy substrates [47,48].

#### 4.1.1. Regulation of Smooth Muscle Contractility

Distinct dietary compounds influence smooth muscle contractility through highly varied mechanisms by interacting with specific receptors located on ENS neurons. Within the murine colonic myenteric plexus, GABAA receptors are composed of multiple subunits, including α1–5 and γ2, with the α4 and α5 subtypes predominantly found on motor neurons. The binding of GABA to these receptors facilitates the release of NO, thereby substantially inhibiting the contractility of the longitudinal smooth muscle [84]. Notably, the expression of the α3 subunit increases with age, reaching its peak in 18-month-old mice, which results in a decreased sensitivity to the contractility-inhibiting effects of GABA in older mice [85]. In an acute stress model, the GABAA receptor positive allosteric modulator alprazolam counteracted stress-induced increases in colonic contractions, an effect that was dependent on the activity of NOS [84].

SCFAs, particularly butyrate, are essential energy substrates for ENS neurons, meeting over 70% of their energy demands. Upon uptake through the monocarboxylate transporter MCT1 into myenteric plexus neurons, butyrate undergoes β-oxidation to produce ATP, thereby maintaining the neuronal capacity to regulate smooth muscle function [73]. Research indicates that intracolonic perfusion of butyrate in neonatal rats leads to an increase in the number of cholinergic neurons (ChAT^+^) within the myenteric plexus, enhances the efficiency of neuromuscular transmission, and improves smooth muscle contractility [86]. Moreover, butyrate significantly increases the proportion of cholinergic neurons through a mechanism mediated by monocarboxylate transporter 2 (MCT2), as silencing MCT2 with siRNA negates the pro-proliferative effects of butyrate on ChAT^+^ neurons [87]. Conversely, *Lactobacillus rhamnosus* interacts with ENS neurons through its surface adhesion protein, leading to the activation of neuronal formyl peptide receptor 1 (FPR1). This activation induces the production of reactive oxygen species (ROS), augments neuronal excitability, and indirectly facilitates smooth muscle contraction. Consequently, this mechanism elevates GI peristaltic frequency in mice by approximately 18%, an effect that can be entirely suppressed by FPR1 antagonists [88].

#### 4.1.2. Maintenance of Peristaltic Frequency

The stability of intestinal motility frequency is dependent on the rhythmic firing activity of ENS neurons and the pacemaker function of interstitial cells of Cajal, which are responsible for generating slow-wave potentials. Dietary components can sustain intestinal motility homeostasis by modulating these two critical elements. Research indicates that the expression of GABAA receptor subunits in the mouse colon exhibits stage-dependent variations throughout development. Notably, the expression of the α1, α2, α5, and γ2 subunits peaks at postnatal day 10 (P10), while α2 and γ2 subunits remain highly expressed at postnatal day 60 (P60). At both developmental stages, GABA significantly enhances colonic peristaltic frequency. However, this regulatory effect is substantially reduced or absent at postnatal day 15 (P15), attributed to a temporary downregulation of the α3 subunit, and in 18-month-old mice due to its excessive upregulation [85]. The modulation of the ENS by GABA presents a multifaceted scenario, with its effects varying across different age groups. This variability is attributed to the dynamic alterations in the composition of GABA receptor subtypes within the gastrointestinal tract over time. Such age-dependent effects imply that dietary intervention strategies should be tailored specifically for infants, adults, and the elderly. Furthermore, colonization with tryptophan-synthesizing bacteria, such as *Bacillus subtilis R0179*, has been shown to increase colonic 5-HT levels in mice. The neurotransmitter 5-HT activates 5-HT4 receptors located on neurons within the myenteric plexus, facilitating the release of ACh and subsequently increasing the frequency of peristalsis. Exogenous administration of 5-hydroxytryptophan (5-HTP) can replicate this effect, thereby alleviating symptoms of constipation [89,90]. SCFAs, which are primary metabolites produced by gut microbiota, have been shown to promote neuronal recovery following antibiotic-induced damage, highlighting their capacity to stimulate enteric neurogenesis in vivo [91]. Additionally, maternal gut dysbiosis prior to conception results in reduced SCFA levels in the offspring’s colon, which suppresses GPR41 signaling and leads to dysfunction of interstitial cells of Cajal pacemakers, manifesting as decreased motility frequency. These adverse effects can be mitigated through butyrate supplementation [92]. Furthermore, prolonged adherence to a diet devoid of fiber, such as one lacking guar gum, diminishes ACh release from cholinergic neurons in the rat myenteric plexus, thereby reducing motility frequency. The introduction of guar gum into the diet restores neuronal signaling and enhances colonic peristalsis [93].

### 4.2. Maintenance of ENS Neuronal Survival and Network Integrity

The preservation of ENS neurons and the structural integrity of the enteric plexuses, specifically the myenteric and submucosal plexuses, are essential for their physiological functionality. Dietary supplementation has been shown to mitigate neuronal loss and sustain plexus density, thereby playing a crucial role during both developmental and aging stages.

#### 4.2.1. Provision of Metabolic Energy for Neurons

Butyrate functions as a crucial energy substrate for ENS neurons, and an inadequate metabolic supply of butyrate may result in neuronal dysfunction. Research has demonstrated that myenteric neurons in the neonatal rat colon exhibit a significantly greater uptake efficiency for butyrate compared to other short-chain fatty acids, with uptake rates approximately three times higher. Intracolonic perfusion of butyrate significantly elevates intracellular ATP levels in neurons and mitigates apoptosis induced by energy failure, underscoring its vital role in maintaining energy homeostasis within the ENS [86].

#### 4.2.2. Inhibition of Inflammatory Damage

Intestinal inflammation can inflict direct damage on ENS neurons, whereas certain dietary components exhibit neuroprotective properties by modulating immune-inflammatory responses. Research indicates that GABA, released by GABAergic neurons in the gut, can activate Gabbr1/2 receptors, thereby inhibiting the proliferation of group 3 innate lymphoid cells (ILC3s) and the secretion of interleukin-17A, ultimately mitigating local inflammation [94]. Additionally, SCFAs, such as butyrate and propionate, in the murine colon activate myenteric plexus neurons via the GPR43 receptor, leading to the release of the anti-inflammatory cytokine IL-10. IL-10 subsequently acts on lamina propria macrophages, suppressing their production of the pro-inflammatory cytokine TNF-α and diminishing the neurotoxic effects of inflammatory factors on the ENS. Supplementation with SCFAs in microbiota-depleted mice has been shown to significantly reduce the expression of inflammatory stress markers, such as inducible nitric oxide synthase (iNOS), in neurons [95].

#### 4.2.3. Maintenance of Plexus Architecture

The structural integrity of the ENS is contingent upon the maintenance of neuronal populations and synaptic connectivity. Dietary components can indirectly enhance the morphology and functionality of enteric plexuses by optimizing the intestinal microenvironment. Studies have demonstrated that antibiotic administration in juvenile mice results in a disruption of gut microbiota, accompanied by a reduction in neuronal numbers within the colonic myenteric plexus. Colonization with *Lactobacillus rhamnosus* has been shown to effectively counteract microbiota disruption, exhibiting more significant neuroprotective effects in male mice [96]. Research has demonstrated that essential vitamins, such as vitamin D, have the capacity to modulate enteric neurons. For example, supplementation with vitamin D has been shown to confer protection against enteric neuronal loss induced by a high-fat diet and palmitic acid in mice. This protective effect is mediated through the attenuation of oxidative stress [97,98].

### 4.3. Modulation of Neuro-Immune Interactions

The ENS serves as a “neuro-immune communication hub” within the GI tract, playing a pivotal role in regulating immune responses to prevent excessive inflammation or immunosuppression. This regulation is achieved through signaling interactions with immune cells, including ILC3s, plasmacytoid dendritic cells (pDCs), and macrophages. Dietary components contribute to the maintenance of intestinal immune homeostasis by modulating these neuro-immune interactions.

The mechanism through which GABA regulates ILC3s is highly conserved across species, with a comparable “neuron-epithelium” axis pathway observed from Caenorhabditis elegans to mammals. In Caenorhabditis elegans, GABA released by enteric neurons targets intestinal smooth muscle via the excitatory receptor EXP-1, leading to the secretion of the neuropeptide FLP-6. Subsequently, FLP-6 acts on intestinal epithelial cells in a paracrine fashion, inhibiting the transcription factor ZIP-10/KLF-1 and activating the PMK-1/p38 MAPK signaling pathway. This activation enhances the expression of antimicrobial peptides, thereby fortifying innate immune defense within the gut [99]. Conversely, enteroendocrine cells are capable of detecting bacterial metabolites of tryptophan, such as indole-3-propionic acid, which stimulate the release of 5-HT through activation of the TRPA1 ion channel. Serotonin, in turn, activates cholinergic neurons, thereby enhancing intestinal motility and indirectly supporting barrier integrity and immune regulation [100].

In synthesizing the evidence presented in this chapter, it becomes apparent that despite the diverse mechanisms of action among various dietary components, their modulation of the ENS follows several overarching principles. Whether through the energetic support provided by SCFAs, the neuromodulatory effects of GABA, or the antioxidant properties of polyphenols, the core outcome converges on the maintenance of ENS homeostasis—namely, ensuring neuronal survival, stabilizing neuro-immune communication, and mitigating oxidative damage. However, the realization of these beneficial effects is not universal but is profoundly influenced by the specific physiological context of the host. Factors such as age, baseline gut microbiota composition, and the local inflammatory milieu can significantly alter the ultimate impact of a given dietary component. Further complexity arises from the dose-dependent effects of many compounds, such as butyrate, which exerts neuroprotective actions at physiological concentrations but may inhibit neuronal function at supra-physiological levels. Currently, research in this field relies heavily on highly controlled animal experiments. While such models are invaluable for elucidating discrete mechanisms, they inevitably fall short in recapitulating the complex reality of the human gut lumen, where multiple dietary components coexist and engage in synergistic or competitive interactions. Thus, the pivotal challenge for future research lies in translating these well-defined mechanisms into effective interventional strategies applicable to the intricate environment of the human body.

## 5. Therapeutic Potential of Dietary Components in ENS-Related Disorders

When the function of the ENS is compromised, it may initiate or worsen a range of local and systemic diseases. The “Pathology” section of Figure 3 provides a systematic summary of the ENS-related diseases examined in this article, categorizing them into three primary groups: intestinal dysfunction, gut–brain axis disorders, and other associated systemic diseases. This classification enhances our understanding of the extensive potential of dietary interventions, and the subsequent sections will provide a detailed examination of the evidence pertaining to each disease category.

### 5.1. Gut-Related Disorders

GI disorders, such as functional constipation and chronic colitis, are typified by pathological characteristics including the loss of ENS neurons, neurotransmitter imbalances, and inflammation-induced neural damage. Dietary compounds have the potential to intervene by modulating ENS signaling, attenuating neuroinflammation, and restoring intestinal motility.

#### 5.1.1. Functional Constipation

Key pathological changes in the ENS associated with functional constipation predominantly include a reduction in neuronal density within the myenteric plexus, dysfunction of nitrergic neurons, and delayed intestinal motility. Dietary interventions may alleviate these pathological conditions by providing energy substrates for the ENS and modulating neurotransmitter signaling. A study conducted on 460 Chinese women with varying bowel movement frequencies employed metagenomic sequencing to analyze gut microbiota and measured serum SCFA levels. The study revealed a positive correlation between the abundance of the butyrate-producing bacterium *Fusobacterium varium* and bowel movement frequency, while serum butyrate concentration was significantly negatively correlated with bowel movement frequency. Further in vitro experiments indicated that low concentrations of butyrate (0.5 mM) promoted enteric neuronal proliferation, whereas high concentrations had inhibitory effects, suggesting a concentration-dependent role of butyrate in the pathogenesis of constipation [101]. Furthermore, the findings suggest that the regulation of the ENS by butyrate exhibits a distinct dose–response relationship, highlighting the importance of precise dosing in its therapeutic application.

In a model of slow-transit constipation, supplementation with a specific probiotic mixture—comprising *Bifidobacterium bifidum W23*, *Bifidobacterium lactis W51*, *Bifidobacterium lactis W52*, *Bifidobacterium longum W108*, *Lactobacillus casei W79*, *Lactobacillus plantarum W62*, and *Lactobacillus rhamnosus W71*—was found to ameliorate constipation phenotypes through multiple mechanisms. This probiotic combination upregulates the c-Kit/SCF signaling pathway, enhances the survival of interstitial cells of Cajal, and inhibits their apoptosis. Additionally, it upregulates TPH1 expression, thereby increasing the biosynthesis of 5-HT and inhibiting its degradation, which promotes intestinal peristalsis. Moreover, the intervention modulated the mRNA and protein expression levels of NOS1, BDNF, TRPV1, and GDNF, suggesting a comprehensive enhancement of enteric nervous system function [102]. While probiotics demonstrate potential in modulating intestinal motility and ENS function, it is crucial to acknowledge that their effects are not universally applicable. Probiotic strains that are effective in animal models may produce inconsistent outcomes in human clinical trials, emphasizing the need for personalized probiotic therapies in future research.

#### 5.1.2. Colitis

In chronic colitis, exemplified by IBD, the excessive release of intestinal inflammatory mediators can result in damage to both ENS neurons and EGCs. Dietary compounds have the potential to mitigate inflammatory responses and safeguard neural structures by modulating the “microbiota–immune–ENS” network.

In a TNBS-induced colitis model, *Akkermansia muciniphila* demonstrated protective effects by decreasing intestinal permeability and inhibiting the NF-κB signaling pathway. This was evidenced by reduced myeloperoxidase activity, a decrease in goblet cell numbers, and lower levels of pro-inflammatory cytokines. Additionally, *Akkermansia muciniphila* alleviated intestinal neuroinflammation and glial cell hyperplasia [103]. In the same model, butyrate supplementation mitigated the loss of nNOS^+^, ChAT^+^, and GPR41^+^ neurons in the colon and reduced the number of GFAP^+^ glial cells. Further analysis indicated that GPR41 co-localized with nitrergic (nNOS^+^) and cholinergic (ChAT^+^) neurons, but not with GFAP^+^ glial cells, suggesting that butyrate primarily exerts its effects directly on enteric neurons via the GPR41 receptor to alleviate colitis-associated neural injury, rather than through glial pathways [104]. Another study found that a 5-HT7 receptor antagonist reduced neural innervation in the mucosal plexus of an IBS-like mouse model by inhibiting the overproduction of neurotrophic factors in the submucosal plexus, highlighting the significant role of 5-HT signaling in the regulation of enteric neuroinflammation [105].

### 5.2. Gut–Brain Axis-Related Disorders

In disorders associated with the gut–brain axis, including PD, spinal cord injury, and depression, the ENS may function either as a locus for the initiation of pathological processes (such as the abnormal aggregation of α-synuclein in PD) or as a peripheral effector in response to central nervous system injury (such as intestinal dysmotility following spinal cord injury). Dietary compounds have the potential to exert therapeutic benefits by maintaining the structural integrity of the ENS and inhibiting the propagation of pathological signals.

#### 5.2.1. PD

A prominent mechanistic hypothesis suggests that pathologically misfolded α-synuclein may initially aggregate within susceptible enteric neurons. This aggregation is proposed to propagate in a prion-like fashion, ascending from the gastrointestinal tract to the brainstem and eventually reaching the substantia nigra through the vagus nerve and other neural pathways. Therefore, dietary interventions aimed at reducing oxidative stress or glial activation in the enteric nervous system may not only alleviate local symptoms but also target the initial or early propagation stages of the fundamental pathological process in Parkinson’s disease. In a rotenone-induced mouse model of PD, there was a significant elevation in intestinal levels of ROS, which was associated with a reduction in tyrosine hydroxylase-positive (TH^+^) dopaminergic neurons in the myenteric plexus and the accumulation of α-synuclein within ENS neurons. Supplementation with chlorogenic acid (CGA, 50 mg/kg) resulted in the upregulation of metallothionein (MT-1/2) expression in both astrocytes and EGCs. MT-1/2, functioning as endogenous antioxidant proteins, directly scavenged ROS and inhibited abnormal α-synuclein aggregation. In vitro experiments further corroborated that pretreatment with CGA significantly enhanced the survival of primary enteric neurons exposed to rotenone and induced the upregulation of MT expression [106].

#### 5.2.2. Spinal Cord Injury

In cases of spinal cord injury (SCI), the primary CNS lesion impairs descending autonomic regulation, resulting in unopposed sympathetic activity and diminished vagal influence on the gastrointestinal tract. This disruption in ‘top-down’ autonomic control directly contributes to ENS dysfunction and subsequent intestinal dysmotility. The aforementioned content has been incorporated into the revised version [107]. Research indicates that dietary supplementation with inulin can prevent ENS atrophy and dysmotility in mice with spinal cord injuries, as demonstrated by a marked restoration in the expression of the pan-neuronal marker PGP9.5 and the nitrergic neuronal enzyme (nNOS). Additionally, interventions involving microbiota-derived SCFAs have been shown to mitigate ENS dysfunction following spinal cord injury. Importantly, the neuroprotective effects of both inulin and SCFAs are contingent upon the activation of the IL-10 signaling pathway [108].

#### 5.2.3. Depression

In murine models of depression, a reduction in central 5-HT levels is frequently accompanied by disrupted 5-HT signaling within the ENS, which is evidenced by a decrease in colonic peristaltic frequency and diminished neuronal activity in the myenteric plexus. In a model of depression induced by chronic unpredictable stress, the expression of tryptophan hydroxylase 1 (TPH1)—the enzyme crucial for 5-HT synthesis—was markedly downregulated in intestinal EC cells, resulting in a decreased colonic 5-HT concentration. This downregulation led to impaired activation of 5-HT3 receptors on ENS sensory neurons and weakened peristaltic reflexes [109]. Supplementation with sustained-release 5-hydroxytryptophan (5-HTP, 10 mg/kg) provided a continuous supply of precursor for 5-HT synthesis in EC cells, thereby restoring local colonic 5-HT levels and activating 5-HT receptors in the myenteric plexus. This activation facilitated the release of ACh and increased the frequency of colonic motility. This intervention not only enhanced intestinal motor function but also ameliorated GI dysfunction associated with depression [109]. In conclusion, dysfunction of the ENS in depression affects the brain through specific pathways. Reduced production of gut-derived 5-HT diminishes vagal signaling to the brain, thereby impairing the transmission of essential homeostatic signals. Additionally, the accompanying intestinal dysfunction induces a state of systemic inflammation. The resultant circulating cytokines have the capacity to directly penetrate the brain, causing damage to normal neurons and aggravating depressive symptoms. Consequently, dietary interventions targeting the ENS may partially ameliorate these disrupted pathways.

### 5.3. Other Related Disorders

#### 5.3.1. Diabetes

In a rat model of diabetes, there was a significant reduction in the total number of neurons (HuC/D^+^) and nitrergic neurons (nNOS^+^) within the ileal myenteric plexus. This neuronal loss was associated with increased levels of lipid peroxidation products, indicating that oxidative stress may play a contributory role in the damage to the ENS [110,111].

Research has demonstrated that administering quercetin microcapsules at a dosage of 10 mg/kg effectively mitigates the loss of nitrergic neurons and significantly reduces oxidative damage in diabetic rats [110]. Conversely, a separate study employing the same model revealed that a higher quercetin dosage (100 mg/kg) diminished the density of interstitial cells of Cajal in the jejunum of healthy rats, suggesting that elevated doses of this compound should be avoided in non-pathological conditions [111]. Therefore, the neuroprotective properties of polyphenols do not consistently exhibit a linear relationship. The efficacy of quercetin is significantly influenced by both dosage and the specific pathological context, necessitating careful consideration before advocating its use as a universal dietary recommendation. Additionally, resveratrol supplementation at 10 mg/kg was found to enhance systemic antioxidant capacity by increasing superoxide dismutase (SOD) activity and glutathione (GSH) levels, thereby neutralizing ROS. It also inhibited the expression of iNOS, which reduced the loss of HuC/D^+^ neurons in the myenteric plexus and improved intestinal motility [112]. In a type 2 diabetic murine model, prebiotic intervention was observed to modulate the gut microbiota and affect enteric neuronal function, thereby inhibiting duodenal hypercontractility and ultimately ameliorating hyperglycemia. This process was contingent upon the synthesis of the bioactive lipid 12-hydroxyeicosatetraenoic acid and involved signaling through mu-opioid receptors located on enteric neurons [113].

#### 5.3.2. Intestinal Ischemia–Reperfusion (I/R)

In a rat model of intestinal I/R injury, there was a significant reduction in the density of HuC/D^+^ neurons within the ileal myenteric plexus. Additionally, nNOS^+^ neurons displayed hypertrophic morphology, and there was a marked increase in the number of S100^+^ EGCs, indicating structural abnormalities in the ENS and heightened glial cell reactivity [114,115]. Pretreatment with resveratrol (10 mg/kg), administered from four days prior to surgery until seven days post-surgery, effectively ameliorated I/R-induced morphological abnormalities in nitrergic neurons and inhibited the hyperproliferation of EGCs [114]. Furthermore, another study demonstrated that oral administration of resveratrol enhanced the reduced/oxidized GSH ratio, improved antioxidant capacity, and facilitated the clearance of accumulated lipid peroxides during I/R injury. These effects significantly contributed to the recovery of HuC/D^+^ neuronal density, thereby corroborating the neuroprotective effects of resveratrol through the alleviation of oxidative stress [115].

The studies reviewed in this chapter unequivocally illustrate the therapeutic potential of dietary interventions for various ENS-related disorders, while concurrently highlighting a significant translational gap between foundational research and clinical application. In animal models of constipation, colitis, and Parkinson’s disease, substantial and promising proof-of-concept data have been obtained. However, rigorously controlled clinical trials in human subjects remain relatively limited, leaving the true therapeutic efficacy of these interventions yet to be conclusively established. This translational challenge arises from multiple factors. Firstly, human diseases such as IBD and PD typically present with complex and chronic etiological characteristics that are challenging to fully replicate using the acute or genetically simplified animal models commonly employed in laboratory research. Secondly, a particularly notable challenge is individual variation. For example, probiotic combinations that demonstrate efficacy in animal studies often produce variable outcomes in human applications, likely due to the unique gut microecological environment and ENS phenotype of each individual. These findings collectively suggest a need for further investigation into personalized approaches and more sophisticated models to bridge the translational divide.

## 6. Conclusions and Future Prospects

Upon thorough examination of extensive evidence, it is evident that despite the diversity of dietary components and ENS-related pathologies, a unifying therapeutic approach has emerged. This approach involves maintaining ENS homeostasis by mitigating oxidative stress, supporting energy metabolism, and modulating neuro-immune communication, thereby representing a potential mechanism through which diet exerts its influence on both gut and brain health.

While preclinical studies in rodent models offer compelling evidence and robust mechanistic insights into the dietary modulation of the ENS, it is crucial to underscore that, from a clinical standpoint, this research area remains nascent. The therapeutic potential discussed herein is predominantly derived from animal models, and a significant translational gap persists in applying these findings to human contexts. One major challenge is the complex bioavailability of dietary components, which significantly restricts their capacity to reach and exert effects on the ENS. For example, polyphenols such as quercetin and resveratrol undergo first-pass metabolism following oral ingestion, resulting in rapid breakdown and elimination during intestinal absorption and hepatic metabolism. Consequently, the concentrations that enter systemic circulation and act on the ENS are substantially lower than the effective concentrations demonstrated in vitro. Additionally, probiotics encounter obstacles such as surviving gastric acid and bile salts and successfully colonizing the intricate and competitive gut ecosystem. The complex processes of absorption, metabolism, and excretion result in a discrepancy between the ingested amount of a substance and the quantity that ultimately exerts an effect at the target site. This discrepancy poses a significant challenge in translating simple dietary supplementation into reliable therapeutic interventions. Furthermore, the extrapolation of dosages from animal models to humans is currently based on empirical estimations, which introduces considerable uncertainty and potential risks. The prevalent approach in current research is to use the body surface area normalization method to convert effective doses from mice to human equivalent doses. However, this method fails to account for critical interspecies differences, such as variations in gut transit time, gut microbiota composition, receptor distribution, and overall metabolic rate. For instance, the concentration of butyrate that exhibits neuroprotective effects in rodents may be challenging to replicate in the human colon through local administration. Similarly, doses of polyphenols that are deemed safe and effective in mice may impose unforeseen burdens on the liver and kidneys with prolonged human consumption. This uncertainty in dose conversion is a primary factor contributing to the failure of many dietary interventions that demonstrate significant effects in animal studies to translate effectively in human clinical trials.

Current research on the ENS continues to encounter substantial limitations. Primarily, the majority of studies are conducted using rodent models, which differ from humans in anatomical structure, cell types, and neural circuit composition, thus constraining the clinical translatability of the results. Furthermore, the high cellular heterogeneity and intricate regulatory networks of the human ENS remain incompletely understood, particularly concerning the dynamic interactions between the ENS and the immune system or microbiota under pathological conditions. Additionally, many studies concentrate on individual dietary components or mechanisms, lacking comprehensive integrative multi-omics analyses, which impedes a holistic understanding of dietary interventions. Lastly, precise nutritional strategies targeting the ENS in specific disease contexts, such as PD or IBD, are still in their nascent stages, with limited clinical evidence regarding personalization, dosage optimization, and long-term safety.

Future research endeavors should prioritize the development of in vitro models that more accurately replicate human physiological characteristics. This includes the utilization of human-derived organoids, neuron-glia co-culture systems, and cross-species comparative research platforms. The application of advanced technologies, such as single-cell sequencing and spatial transcriptomics, is essential for a comprehensive analysis of the cell types, signaling pathways, and network functions of the ENS under both normal and pathological conditions. Moreover, in the context of ENS injury, the sequential administration of various dietary components may result in more pronounced neuroprotective and functional recovery outcomes compared to the simultaneous or individual administration of these components. Utilizing human ENS single-cell RNA sequencing data facilitates the identification of susceptible neuronal subtypes, such as specific nitrergic neurons, in particular pathologies like Parkinson’s disease. This approach enables the screening of dietary compounds that selectively activate or protect these neuronal subtypes. Furthermore, dietary components can be encapsulated with specific materials, such as pH-sensitive substances, to develop targeted microcapsules. These microcapsules are designed to release high concentrations of dietary substances precisely in critical intestinal regions, thereby improving local bioavailability. It is imperative to acknowledge that the promising potential of dietary interventions for ENS diseases hinges on our capacity to validate these effects in human subjects and to ascertain key parameters, including effective dosage, treatment duration, and variability in individual responses. Furthermore, future research should prioritize longitudinal studies within pediatric and adolescent cohorts. The ENS and the gut–microbiota–brain axis demonstrate considerable plasticity during early life and adolescence, presenting a critical window for preventive and therapeutic interventions. Elucidating the influence of dietary factors on ENS maturation and long-term health during these pivotal developmental stages is crucial for formulating effective nutritional strategies aimed at preventing the onset or mitigating the progression of ENS-related disorders, such as pediatric functional gastrointestinal disorders or early-onset neurodegenerative diseases.

## Data Availability

No new data were created or analyzed in this study. Data sharing is not applicable to this article.

## Abbreviations

ANS: autonomic nervous system; Ach: acetylcholine; AhR: aryl hydrocarbon receptor; CNS: central nervous system; DSS: dextran sulfate sodium; ENS: enteric nervous system; EGCs: enteric glial cells; ECs: enterochromaffin cells; FPR1: formyl peptide receptor 1; GDNF: glial cell line-derived neurotrophic factor; GI: gastrointestinal; GABA: gamma-aminobutyric acid; GAD: glutamate decarboxylase; GSH: glutathione; IBD: inflammatory bowel disease; IFN-γ: interferon-gamma; ILA: indole-3-lactic acid; IAA: indole-3-acetic acid; IL-10: interleukin-10; iNOS: inducible nitric oxide synthase; ILC3s: group 3 innate lymphoid cells; I/R: ischemia–reperfusion; KYN: kynurenine; KYNA: kynurenic acid; MMC: migrating motor complex; MCT1: monocarboxylate transporter 1; nAChRs: nicotinic acetylcholine receptors; NPY: neuropeptide Y; NO: nitric oxide; NOS: nitric oxide synthase; PNS: peripheral nervous system; pDCs: plasmacytoid dendritic cells; PD: parkinson’s disease; QA: quinolinic acid; ROS: reactive oxygen species; SCFAs: short-chain fatty acids; SP: substance P; SMCT1: sodium-coupled monocarboxylate transporter 1; SOD: superoxide dismutase; TPH1: tryptophan hydroxylase 1; VIP: vasoactive intestinal peptide; 5-HT: 5-hydroxytryptamine.
